# P2Y_2_ and P2Y_6_ receptor activation elicits intracellular calcium responses in human adipose-derived mesenchymal stromal cells

**DOI:** 10.1007/s11302-018-9618-3

**Published:** 2018-08-07

**Authors:** Seema Ali, Jeremy Turner, Samuel J. Fountain

**Affiliations:** 1 School of Biological Sciences, University of East Anglia, Norwich Research Park, Norwich, NR4 7TJ UK; 2 Norfolk & Norwich University Hospital, Norwich, NR4 7UY UK; 3 Norwich Medical School, University of East Anglia, Norwich, NR4 7TJ UK

**Keywords:** Adipose tissue, AR-C118925XX, MRS2578, ATP, ADP

## Abstract

Adipose tissue contains self-renewing multipotent cells termed mesenchymal stromal cells. In situ, these cells serve to expand adipose tissue by adipogenesis, but their multipotency has gained interest for use in tissue regeneration. Little is known regarding the repertoire of receptors expressed by adipose-derived mesenchymal stromal cells (AD-MSCs). The purpose of this study was to undertake a comprehensive analysis of purinergic receptor expression. Mesenchymal stromal cells were isolated from human subcutaneous adipose tissue and confirmed by flow cytometry. The expression profile of purinergic receptors was determined by quantitative real-time PCR and immunocytochemistry. The molecular basis for adenine and uracil nucleotide-evoked intracellular calcium responses was determined using Fura-2 measurements. All the known subtypes of P2X and P2Y receptors, excluding P2X2, P2X3 and P2Y_12_ receptors, were detected at the mRNA and protein level. ATP, ADP and UTP elicited concentration-dependent calcium responses in mesenchymal cells (*N* = 7–9 donors), with a potency ranking ADP (EC_50_ 1.3 ± 1.0 μM) > ATP (EC_50_ 2.2 ± 1.1 μM) = UTP (3.2 ± 2.8 μM). Cells were unresponsive to UDP (< 30 μM) and UDP-glucose (< 30 μM). ATP responses were attenuated by selective P2Y_2_ receptor antagonism (AR-C118925XX; IC_50_ 1.1 ± 0.8 μM, 73.0 ± 8.5% max inhibition; *N* = 7 donors), and UTP responses were abolished. ADP responses were attenuated by the selective P2Y_6_ receptor antagonist, MRS2587 (IC_50_ 437 ± 133nM, 81.0 ± 8.4% max inhibition; *N* = 6 donors). These data demonstrate that adenine and uracil nucleotides elicit intracellular calcium responses in human AD-MSCs with a predominant role for P2Y_2_ and P2Y_6_ receptor activation. This study furthers understanding about how human adipose-derived mesenchymal stromal cells can respond to external signalling cues.

**Electronic supplementary material:**

The online version of this article (10.1007/s11302-018-9618-3) contains supplementary material, which is available to authorized users.

## Introduction

Adipose tissue is an abundant and easily accessible source of mesenchymal stromal cells (MSCs) in adult humans. MSCs are multipotent plastic-adherent cells that can be isolated from bone marrow and other tissues, including adipose [1]. In adult adipose tissue, a pool of MSCs serves to replace approximately 10% of mature adipocytes annually via adipogenesis [2]. Both adipogenesis and hypertrophic mechanisms are important for the expansion of adipose tissue and body buffering of glucose and free fatty acids. Lower rates of adipogenesis are associated with increased visceral obesity, adipocyte hypertrophy and higher fasting blood glucose levels [3]. This suggests that efforts to improve the adipogenic potential of MSCs may oppose metabolically unhealthy phenotypes. The multipotency of adipose-derived (AD)-MSCs [1, 4] has led to clinical trial evaluation of AD-MSCs usage for regenerative tissue damage [5–7]. Despite some promise in tissue regeneration studies, MSC senescence and induced inflammation are common drawbacks to therapy [8, 9]. A greater understanding of how MSCs respond to their environment via cell surface receptors will therefore delineate MSC function.

Signalling via extracellular nucleotides has been implicated in cell migration, proliferation, differentiation and inflammation [10, 11]. The biological effects of ATP are mediated by P2X receptors (P2X1-7), a family of trimeric cation ion channels [12, 13], and metabotropic responses to ATP, ADP, UTP, UDP and UDP-sugars are mediated by eight P2Y receptors in a subtype-specific fashion [14]. P2Y_1_, P2Y_2_, P2Y_4_, P2Y_6_ and P2Y_11_ receptors are G_q_-coupled, so upon agonist binding they activate phospholipase C (PLC) and subsequently induce release of calcium (Ca^2+^) from intracellular stores, whereas P2Y_12_, P2Y_13_ and P2Y_14_ receptors are G_i_-coupled and consequently suppress adenylyl cyclase activity [15]. Receptor-mediated intracellular Ca^2+^ signals are known to be important for cellular proliferation and differentiation, and studies have demonstrated stem cell sensitivity to extracellular ATP [16]. However, current work has primarily focused on rodent models and bone marrow-derived MSCs (BM-MSC) or has failed to report the molecular basis of purinergic responses [17, 18]. For example, extracellular nucleotides elicit reorganisation of actin filaments and cell migration in 3T3-L1 mouse adipocyte precursors [19], and ATP promotes adipogenic and osteogenic differentiation in BM-MSCs [20]. This study focuses on the molecular identity of receptors used by human AD-MSCs to respond to physiologically relevant extracellular nucleotides and elevate cytoplasmic calcium.

## Methods

### Chemicals and antibodies

All chemicals were purchased from Sigma-Aldrich (Dorset, UK) unless otherwise stated. Selective antagonists were obtained from Tocris Bioscience (Bristol, UK) (P2Y_1_ MRS2500; P2Y_2_ AR-C118925XX; P2Y_6_ MRS2578; P2Y_11_ NF340; P2Y_12_ PSB-0739; P2Y_13_ MRS2211; P2X4 PSB12062; P2X7 A438079), excluding thapsigargin (sarco-endoplasmic reticulum Ca^2+^-ATPases, SERCA) and U73122 (PLC) (Santa Cruz Biotechnology, Texas, USA). Nucleotides were purchased from Abcam (Cambridge, UK), except ADP (Sigma-Aldrich, Dorset, UK). Primary antibodies were purchased from Santa Cruz Biotechnology (Dallas, TX, USA) (P2X1 sc-31491; P2X5 sc-15192), Alomone Labs (Jerusalem, Israel) (P2X4, APR-002; P2X7, APR-004; P2Y_1_, APR-009; P2Y_4_, APR-006; P2Y_6_, APR-011; P2Y_11_, APR-015; P2Y_12_, APR-020; P2Y_13_, APR-017) and Abcam (Cambridge, UK) (P2Y_2_, ab10270). Phycoerythrin (PE)-conjugated IgG_1_ isotype control (400113), CD14 (367103), CD45 (368509), CD73 (344003), CD90 (328109) and CD105 (323205) antibodies were all purchased from Biolegend (San Diego, CA, USA).

### Tissue donation

Subcutaneous abdominal adipose tissue samples were obtained from 48 healthy female volunteers, who have had mastectomies as part of their past treatment for breast cancer and subsequently choose to have breast reconstructions via elective delayed deep inferior epigastric perforator flap operations. All volunteers were screened to exclude diabetics, current cancer sufferers, patients currently receiving chemotherapy, donors with infections or taking anti-inflammatory medication. The donors had an average age of 55.5 ± 1.4 (range 38–75). Samples were obtained with the assistance of the plastic surgery team at the Norfolk and Norwich University Hospital (NNUH). Informed consent was obtained from all volunteers prior to participation in the study.

This study was ethically approved by the London-Stanmore Research Ethics Committee (152093) and the Research and Development department at the NNUH (2014EC03L).

### Primary cell isolation from adipose tissue

Fresh adipose tissue samples were dissected to remove blood vessels, fibrous tissue and skin. The samples were then further minced and enzymatically digested with collagenase and DNase I for 30 min at 37 °C with regular mixing by inversion. The digested tissue samples were then passed through a 70-μm cell strainer and centrifuged for 5 min at 450×*g*, which separated the sample into a floating fraction containing mature adipocytes and a pellet containing MSCs. The adipocytes were discarded and the MSCs were treated with a red cell lysis buffer, washed and then resuspended in DMEM supplemented with 4.5 g/L glucose, L-glutamine, 10% FBS (*v*/*v*), 50 IU/ml penicillin and 50 μg/ml streptomycin and left in a T175 flask overnight in a humidified incubator at 37 °C in 5% CO_2_. The following day, the cells were washed twice with PBS to remove any non-adherent cells or debris and left in fresh serum-containing media until the cells were confluent, at which point they were trypsinised and plated for experimental use. MSCs were passaged up to eight times for experimental use, but the majority of experiments were performed between passage one and four.

### Flow cytometry

MSCs were trypsinised and resuspended in PBS at a density of 1 × 10^6^ cells/ml and then 100 μl of cells were placed in individual tubes for each marker and control required. All steps were conducted at room temperature. The cells were incubated with 5 μg/ml of human BD Fc block™ (BD Pharmingen, New Jersey, USA) for 10 min. Next, PE-conjugated antibodies (1:33) were added to detect cell surface expression of CD14, CD45, CD73, CD90 and CD105 and the cells were incubated in the dark for a further 30 min. An isotype control (1.33) and an unstained control were also run alongside these markers. The cells were then washed once with PBS and resuspended in 200 μl of fresh PBS. Samples were then analysed using a Beckman Coulter CytoFLEX flow cytometer (California, USA). Fluorescence intensity was read for PE (excitation 496 nm, emission 578 nm). Living cells were gated according to their forward and side scatter and then histograms were plotted to compare the fluorescence signal for each marker versus the isotype control using CytExpert 1.2.11 software (Beckman Coulter, California, USA).

### Calcium mobilisation experiments

MSCs were seeded at 2 × 10^4^ cells per well in black glass bottom 96-well plates (Molecular Devices, California, USA) and incubated at 37 °C in 5% CO_2_ for 48 h. The growth media was then aspirated off and the cells were gently washed with salt buffered solution (SBS) (pH 7.4), containing 130 mM sodium chloride, 5 mM potassium chloride, 1.2 mM magnesium chloride, 1.5 mM calcium chloride, 8 mM D-(+)-glucose and 10 mM HEPES. The cells were then loaded with 2 μM Fura-2AM (TEFLabs, Austin, TX, USA) in SBS supplemented with 0.01% (*w*/*v*) pluronic for 1 h at 37 °C while being protected from light. The loading buffer was then removed and the cells were washed twice with SBS. Where applicable, the cells were incubated for a further 30 min with antagonists/vehicle or calcium-free SBS (SBS lacking 1.5 mM calcium chloride, but containing 2 mM EGTA, pH7.4). All antagonists were dissolved in water or DMSO and were then further diluted in SBS, so that a final concentration of 1% DMSO was never exceeded. Finally, the cells were maintained at 37 °C and challenged with nucleotides administered as a single pipette drop by a FlexStation III microplate reader (Molecular Devices, California, USA), which also recorded the average fluorescence (excitation 340 and 380 nm, emission 510 nm) per well every 3 s to provide *F*_ratio_ values. *F*_ratio_ values at every time point, peak *F* ratios and area under the curve data were extracted using SoftMax Pro 5.4.5 (Molecular Devices, California, USA) software.

### Immunocytochemistry

MSCs were seeded onto glass coverslips and incubated at 37 °C for 48 h. All subsequent steps were conducted at room temperature unless otherwise stated. Culture media was gently aspirated off the cells and the cells were washed with PBS, fixed with 4% paraformaldehyde for 15 min and then permeabilised with 0.25% Triton X-100 for 10 min. Non-specific binding was blocked with 1% bovine serum albumin and then the cells were incubated with the appropriate primary antibody (1:200) overnight at 4 °C. The excess primary antibody was removed and successful binding was detected using rabbit anti-goat (Abcam, Cambridge, UK) or goat anti-rabbit (Thermo Fisher Scientific, Waltham, MA, USA) Alexa Fluor 488-conjugated secondary antibodies (1:1000 dilution). Finally, cells were mounted using VectaShield containing 1.5 μg/ml DAPI (Vector Laboratories, Peterborough, UK) and imaged using a Zeiss AxioPlan 2ie epifluorescent microscope (Carl Zeiss Ltd., Cambridge, UK).

### RNA extraction, cDNA synthesis and quantitative real-time PCR

MSCs were lysed with TRI-reagent and then treated with 100 μl 1-bromo-3-chloropropane and centrifuged to partition the sample into three phases. The top aqueous phase was then carefully transferred into a fresh tube and the RNA was precipitated with isopropanol and washed with 75% ethanol. The RNA was then centrifuged at 12,000×*g* for 10 min, the supernatant was removed and the RNA pellet was air dried. The resultant RNA was then resuspended in molecular grade water and potential genomic DNA contamination was removed using a DNA-free™ DNA removal kit (Thermo Fisher Scientific, Waltham, MA, USA) as per the manufacturer’s instructions. The purity and quantity of RNA was assessed using a Nanodrop 2000 (Thermo Scientific, Delaware, USA).

RNA (500 ng for each sample) was primed with 100 ng random hexamer primers (Bioline, Massachusetts, USA) by heating the mixture to 70 °C for 10 min. Each sample was then incubated with 250 μM dNTPs (Bioline, Taunton, MA, USA), 30 U RNasin ribonuclease inhibitor (Promega, Madison, WI, USA), 0.01 M DTT, first strand buffer and 200 U Superscript III Reverse transcriptase (RT) (Thermo Fisher Scientific, Waltham, MA, USA) for 1 h at 42 °C. A duplicate sample with no RT was run alongside as a control. The PCR reaction was terminated by heating the samples to 70 °C for 10 min. Complementary DNA (cDNA) samples were then stored at − 20 °C.

The cDNA and their corresponding no RT controls were diluted to 2 ng/μL and mixed with TaqMan™ fast universal PCR master mix. Commercially available TaqMan gene expression assay primers and probes for each gene of interest (GOI) were also added (P2Y_1_ Hs00704965_s1; P2Y_2_ Hs04176264_s1; P2Y_4_ Hs00267404_s1; P2Y_6_ Hs00366312_m1; P2Y_11_ Hs01038858_m1; P2Y_12_ Hs01881698_s1; P2Y_13_ Hs03043902_s1; P2Y_14_ Hs01848195_s1; P2X1 Hs00175686_m1; P2X2 Hs04176268_g1; P2X3 Hs01125554_m1; P2X4 Hs00602442_m1; P2X5 Hs01112471_m1; P2X6 Hs01003997_m1; P2X7 Hs00175721_m1; RPLP0 Hs99999902_m1). Each sample was then amplified in a MicroAmp fast optical 96-well reaction plate on an Applied Biosystems 7500 Real-Time PCR System (Thermo Fisher Scientific, Waltham, MA, USA) for 40 cycles. *C*_T_ values were extracted from the 7500 software v2.0.6. Receptors with *C*_T_ values of below 35 were deemed to be expressed. Ribosomal protein lateral stalk subunit P0 (RPLP0) was used as an endogenous control to calculate the ∆*C*_T_ values (∆*C*_T_ = average GOI *C*_T_–average RPLP0 *C*_T_) and therefore normalise for any variability in cDNA template input.

### Statistical analysis

Results were analysed, including statistical analyses, using Origin Pro 2017 software (Origin Lab, Northampton, MA, USA). All concentration response data were normalised to the maximal response. In cases of variability between donors, the concentration that produced the maximum response in the majority of donors was used. All antagonist data were normalised to their respective vehicle controls. Concentration response curves were fitted by Origin using the Hill Equation. The tau values were calculated by fitting single exponential decay curves.

Data were assessed for normality using a Shapiro-Wilk test and then normally distributed data were assessed using a paired/unpaired Student’s *t* test or ANOVA with a post hoc Tukey test. Non-normally distributed data were assessed by paired sample Wilcoxon signed-rank test, Mann-Whitney test or Kruskal-Wallis ANOVA with a post hoc Dunn’s test. Data are expressed as mean ± SEM of experiments performed in duplicate using cells from a minimum of three independent donors.

## Results

### Phenotypic characterisation of human adipose-derived MSCs

**Fig. 1:**
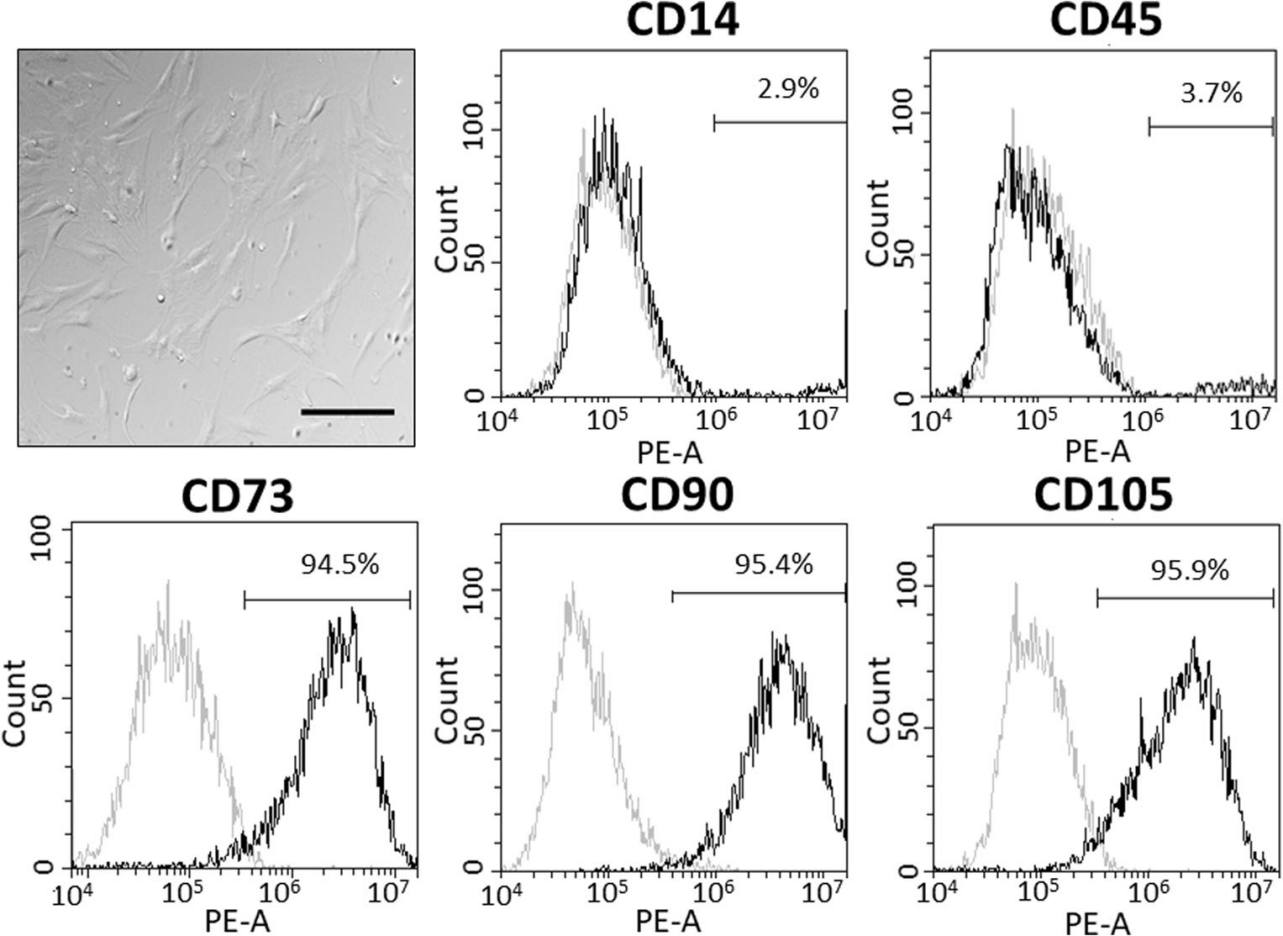
Phenotypic characterisation of human adipose-derived mesenchymal stromal cells by flow cytometry and differential interference contrast microscopy of mesenchymal stromal cells in culture. The scale bar represents 200 μm. Flow cytometric analysis of cell surface marker expression in human mesenchymal stromal cells of known positive (CD73, CD90, CD105) and negative (CD14 and CD45) markers. Grey histogram indicates the isotype control (PE-conjugated anti-IgG1) and the black histogram shows the surface antigen expression level. Data for one representative donor shown and the percentages displayed correspond specifically to this one donor

The cells used in this study were all plastic adherent (Fig. 1) and capable of differentiating to mature adipocytes and osteoblasts when cultured in adipogenic or osteogenic media respectively (Online resource 1). MSCs were not capable of spontaneously differentiating to either cell type, which is consistent with previous findings [21]. MSCs were strongly positive for expected cell surface markers CD73 (90.2 ± 2.5% positivity, *N* = 6 donors), CD90 (89.8 ± 2.9% positivity, *N* = 6 donors) and CD105 (83.7 ± 3.3% positivity, *N* = 6 donors), and expressed CD14 (10.0 ± 4.0% positivity, *N* = 6 donors) and CD45 (11.8 ± 4.3% positivity, *N* = 6 donors) at low levels (Fig. 1). These criteria are in line with the MSC definition outlined by the International Society for Cellular Therapy [1].

### Nucleotides evoked intracellular calcium responses in AD-MSCs

**Fig. 2:**
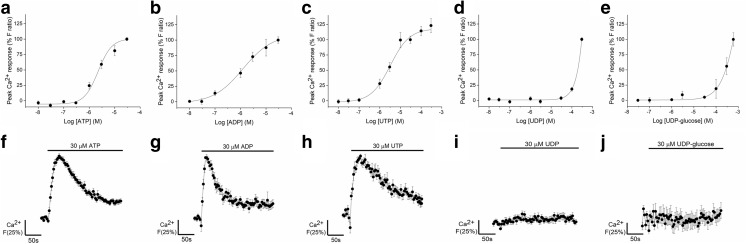
ATP, ADP and UTP elicited intracellular Ca^2+^ responses in human adipose-derived mesenchymal stromal cells (**a–e**) Concentration response curves for the magnitude of intracellular Ca^2+^ responses elicited by (**a**) ATP (*N* = 9 donors), (**b**) ADP (*N* = 8 donors), (**c**) UTP (*N* = 7 donors), (**d**) UDP (*N =* 6 donors) and (**e**) UDP-glucose (*N =* 3 donors). Ca^2+^ responses were normalised to the maximal response observed in the majority of donors, which was the response to 30 μM for ATP, ADP and UTP, 300 μM for UDP and 600 μM for UDP-glucose. Average data for donors that responded to nucleotide stimulation are shown. Data for donors that did not respond to nucleotide stimulation were not included. (**f**–**j**) Averaged time-resolved intracellular Ca^2+^ responses elicited by 30 μM of each agonist. Traces were normalised to the maximal response within a donor and averaged across donors. Data points are mean ± SEM

In the presence of extracellular calcium, ATP, ADP and UTP elicited concentration-dependent increases in intracellular Ca^2+^ in all donors tested (*N* = 7–9 donors) (Fig. 2). These responses were unaffected by multiple passaging of the cells (data not shown). Nucleotides had a rank order of potency ADP (EC_50_ 1.3 ± 1.0 μM) > ATP (EC_50_ 2.2 ± 1.1 μM) = UTP (3.2 ± 2.8 μM) (Fig. 2a–c). All three nucleotides elicited an initial rapid response that decayed to approximately 25% above baseline intracellular Ca^2+^ levels within recording period of 250 s (Fig. 2f–h). Between nucleotides, the magnitude of responses and net Ca^2+^ movement were comparable at maximal concentrations (Table 1), though responses to ADP decayed significantly faster than responses elicited by ATP or UTP (*p* < 0.05, *N* = 7–9 donors; Table 1).

**Table 1:** Characteristics of the calcium responses evoked by maximal concentrations of nucleotides (30 μM) in primary human adipose-derived mesenchymal stromal cells. Mean ± SEM

Nucleotide	Peak magnitude (*F* ratio)	Net calcium movement (area under the curve)	Decay time, τ (s)	EC_50_ (μM)
ATP (N = 9)	0.422 ± 0.05	53.7 ± 5.9	73.4 ± 230.2	2.24 ± 1.1
ADP (*N* = 8)	0.385 ± 0.05	39.6 ± 6.2	35.3 ± 6.3^a^	1.25 ± 1.0
UTP (*N* = 7)	0.334 ± 0.06	41.9 ± 7.8	122.4 ± 4447.5	3.24 ± 2.8

^a^Decay time was significantly faster for the ADP-evoked calcium response vs ATP (*p < 0.005*) and UTP (*p < 0.005*) as determined by Kruskal-Wallis ANOVA with post hoc analysis using Dunn’s test

No responses were detected for 30 μM UDP or below (*N =* 6 donors) (Fig. 2i). However, responses were consistently detected in the presence of 300 μM UDP (*N =* 6 donors), with some donors displaying very small responses (peak *F* ratio 0.12 ± 0.04, *N =* 3 of 6 donors) with the addition of 100 μM UDP (Fig. 2d). Agonist concentrations of above 30 μM UDP are not likely to be representative of physiological nucleotide concentrations, so it is unlikely that these results show true activation of UDP-sensitive receptors. Similarly, exogenous application of UDP-glucose elicited a Ca^2+^ response in some donors (*N =* 3 of 9 donors) (Fig. 2e), but these responses were only evident at very high agonist concentrations of greater than 100 μM (*N* = 1) or 300 μM (*N =* 2). No response was detected for 30 μM UDP-glucose (*N =* 9 donors) (Fig. 2j). Furthermore, six out of a nine donors tested did not display a response to UDP-glucose at any concentration tested (up to 600 μM). The EC_50_ values for both UDP and UDP-glucose could not be accurately calculated as the responses had not plateaued within the range of concentrations tested.

### Metabotropic receptors mediated nucleotide-evoked intracellular calcium responses

**Fig. 3:**
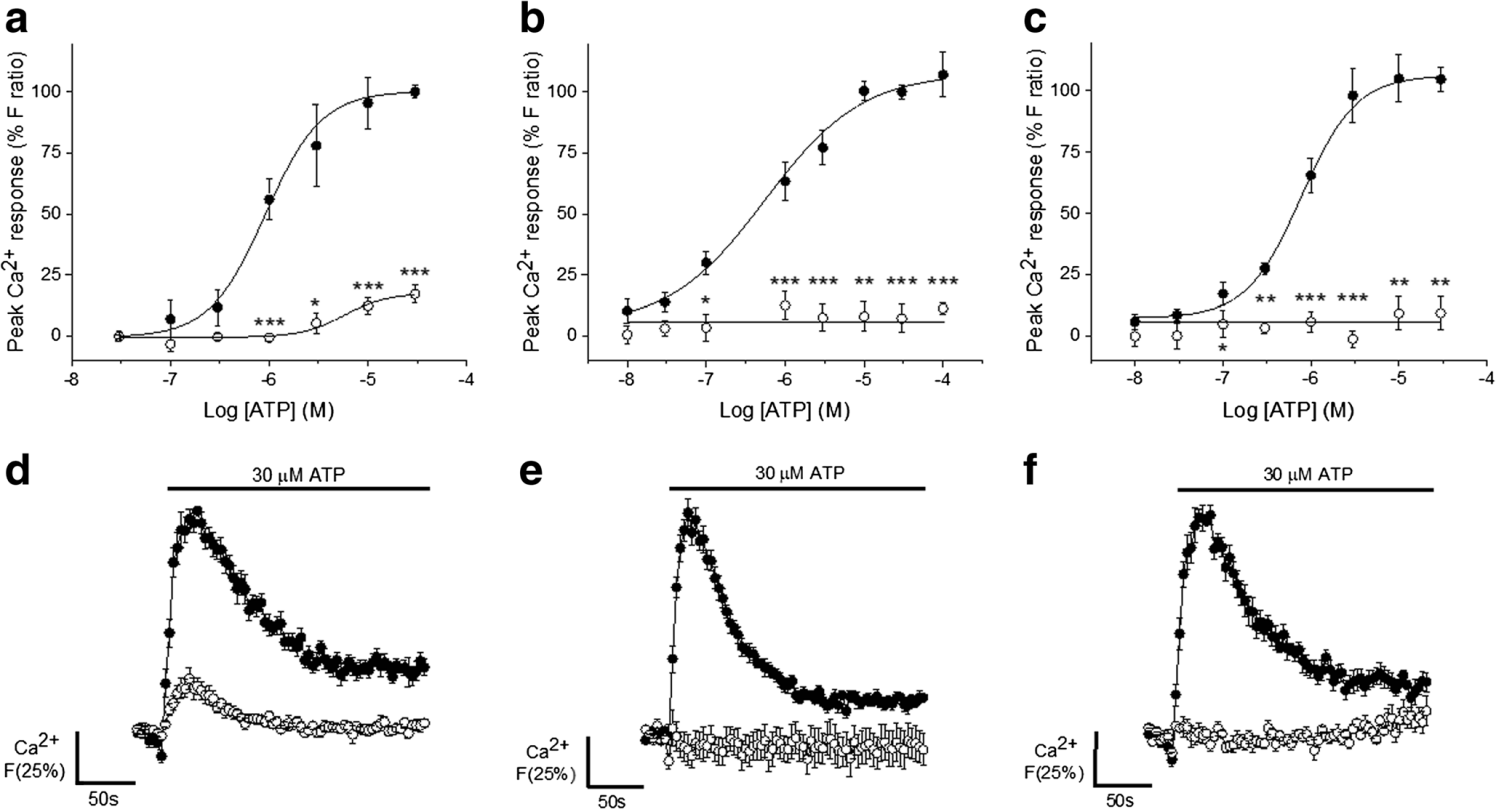
Dependency of ATP-elicited intracellular Ca^2+^ responses on Ca^2+^ influx, PLC activity and release of Ca^2+^ from intracellular stores in human adipose-derived mesenchymal stromal cells. **a** ATP dose-response curve for intracellular Ca^2+^ responses in the presence (*closed circles*) and absence (*open circles*) of 1.5 mM extracellular Ca^2+^ (*N* = 6). **b** ATP dose-response curve under control conditions (*closed circles*) or following phospholipase C inhibition (10 μM U73122) (*N* = 6). **c** ATP dose-response curve under control conditions (*closed circles*) or following sarco-endoplasmic reticulum Ca^2+^-ATPases inhibition induced emptying of the intracellular Ca^2+^ stores (5 μM thapsigargin) (*open circles*) (*N* = 6). **d** Average time-resolved trace showing responses elicited by 30 μM ATP in the presence (*closed circles*) and absence *(open circles*) of 1.5 mM extracellular Ca^2+^ (*N* = 6). **e** Average time-resolved traces for the response to 30 μM ATP under control conditions (*closed circles*) and following U73122 treatment (*open circles*) (*N* = 6). **f** Average time-resolved traces for the response to 30 μM ATP under control conditions (*closed circles*) and following thapsigargin treatment (*open circles*) (*N* = 6). Data points are mean±SEM. *p < 0.05, **p < 0.01, ***p < 0.001

**Fig. 4:**
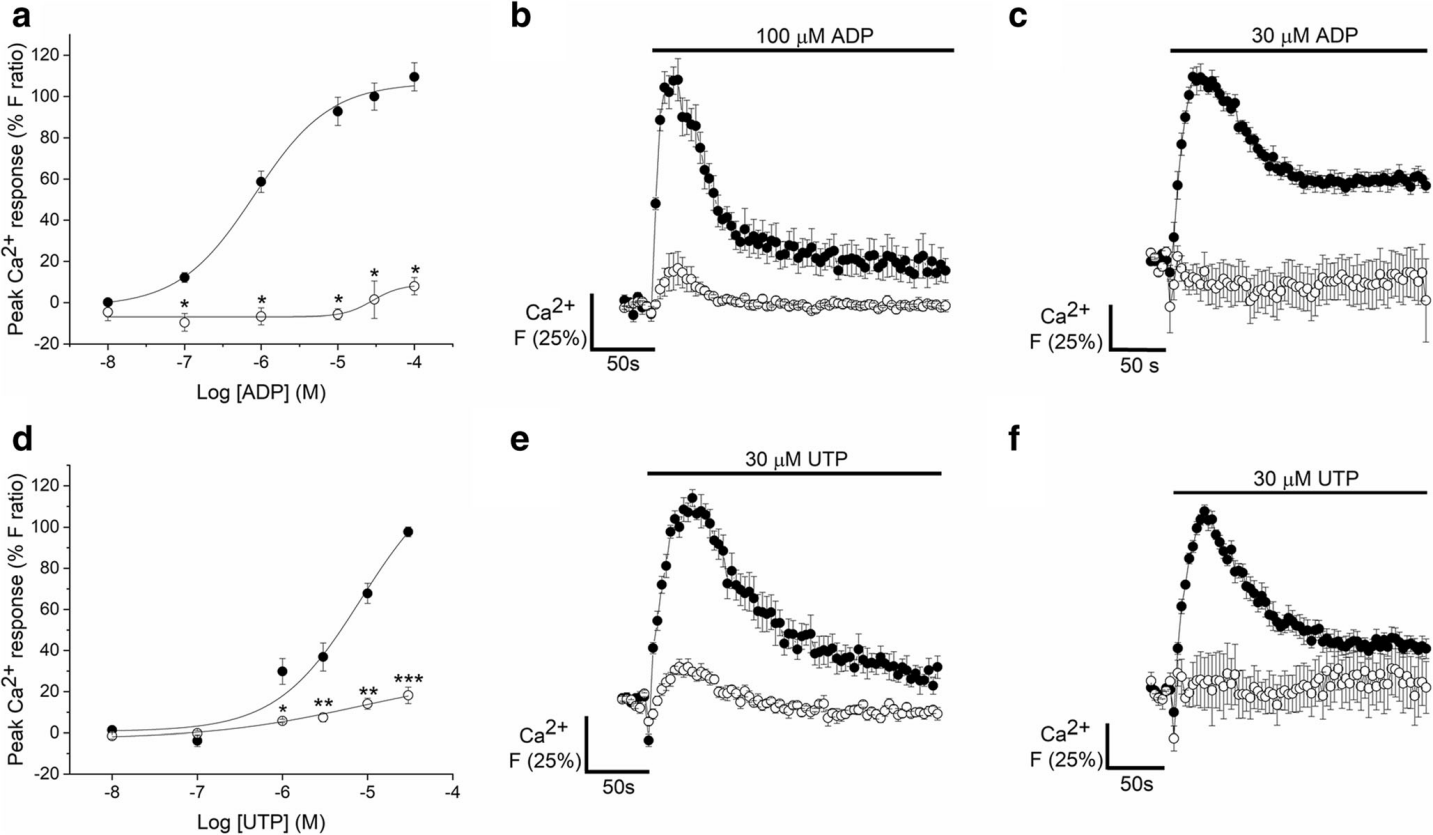
Dependency of ADP- and UTP-elicited intracellular Ca^2+^ responses on Ca^2+^ influx and PLC in human adipose-derived mesenchymal stromal cells. **a** ADP dose-response curve for intracellular Ca^2+^ responses in the presence (*closed circles*) and absence (*open circles*) of 1.5 mM extracellular Ca^2+^ (*N* = 3). **b** Average time-resolved trace showing responses elicited by 100 μM ADP in the presence (*closed circles*) and absence *(open circles*) of 1.5 mM extracellular Ca^2+^ (*N* = 3) **c** Average time-resolved traces for the response to 30 μM ADP under control conditions (*closed circles*) and following treatment with 10 μM U73122 (*open circles*) (*N* = 4). **d** UTP dose-response curve for intracellular Ca^2+^ responses in the presence (*closed circles*) and absence (*open circles*) of 1.5 mM extracellular Ca^2+^ (*N* = 4). **e** Average time-resolved trace showing responses elicited by 30 μM UTP in the presence (*closed circles*) and absence *(open circles*) of 1.5 mM extracellular Ca^2+^ (*N* = 4) **f** Average time-resolved traces for the response to 30 μM UTP under control conditions (*closed circles*) and following U73122 treatment (*open circles*) (*N* = 3). Data points are mean ± SEM. *p < 0.05, **p < 0.01, ***p < 0.001

The responses elicited by maximal ATP, ADP and UTP concentrations persisted, but were decreased by 82.7 ± 3.5% (*N* = 6, *p* < 0.001) (Fig. 3a, d), 92.0 ± 4.2% (*N* = 3, *p* < 0.05) (Fig. 4a–b) and 81.8 ± 4.0% (*N* = 4, *p* < 0.001) (Fig. 4d–e), respectively, following removal of Ca^2+^ from the extracellular solution with 2 mM EGTA. The nucleotide-evoked responses also all returned to baseline Ca^2+^ levels within the sampling period when extracellular Ca^2+^ was removed, instead of remaining approximately 25% above baseline. In addition, in the absence of extracellular calcium, ATP-evoked responses decayed faster (τ 31.2 ± 5.4 s without Ca^2+^ vs τ 55.0 ± 3.6 s with Ca^2+^, *p <* 0.005; *N* = 6 donors) and although the responses to ADP and UTP displayed the same trend, the respective alterations in the decay times were not statistically significant (Table 2). Also, despite the appearance of a rightward shift in the concentration response curves (Fig. 3a, 4a, d), variation between donors meant that the EC_50_ values were not significantly altered for any of the nucleotides when extracellular calcium was removed (Table 2). Furthermore, inhibition of PLC, which is part of the downstream signalling pathway instigated upon Gq-coupled P2Y receptor activation, with U73122 abolished the responses to ATP (*N =* 6) (Fig. 3b, e), ADP (*N =* 4) (Fig. 4c) and UTP (*N =* 3) (Fig. 4f) respectively. Also, depleting the endoplasmic reticulum Ca^2+^ stores by inhibiting sarco-endoplasmic reticulum Ca^2+^-ATPases (SERCA) with thapsigargin (*N =* 6) (Fig. 3c, f) abolishes the response to ATP. Together, these data suggest that the nucleotide responses in human AD-MSCs are mediated by metabotropic receptors and that the magnitude of the response, as well as the sustained Ca^2+^ elevation, may be dependent upon extracellular Ca^2+^ influx.

**Table 2:** Changes in the magnitude, decay kinetics and potency of nucleotide-evoked calcium responses in primary human adipose-derived mesenchymal stromal cells in the presence and absence of extracellular calcium, [Ca^2+^]_e_. Mean ± SEM

Nucleotide	Peak magnitude (*F* ratio)^a^		Decay time, τ (s)		EC_50_ (μM)	
	+ [Ca^2+^]_e_	− [Ca^2+^]_e_	+ [Ca^2+^]_e_	− [Ca^2+^]_e_	+ [Ca^2+^]_e_	− [Ca^2+^]_e_
30 μM ATP (*N* = 6)	0.68 ± 0.1	0.11 ± 0.03	55.0 ± 3.6	31.2 ± 5.4^b^	0.9 ± 0.4	5.7 ± 2.6
100 μM ADP (*N* = 3)	1.08 ± 0.1	0.17 ± 0.1	30.3 ± 1.4	22.5 ± 72.4	0.8 ± 0.6	28.3 ± 62.1
30 μM UTP (*N* = 4)	0.57 ± 0.04	0.12 ± 0.02	59.7 ± 27	41.2 ± 5.9	8.3 ± 1.4	7.8 ± 2.8

^a^The peak magnitude was significantly decreased when extracellular calcium is removed for ATP (*p* < 0.001), ADP (*p* < 0.05) and UTP (*p* < 0.001) as determined by paired *T* tests or Wilcoxon signed-rank test

^b^The decay time was significantly faster for the ATP-evoked calcium response in the absence of extracellular calcium (*p < 0.005*) as determined by a paired *T* test

**Fig. 5:**
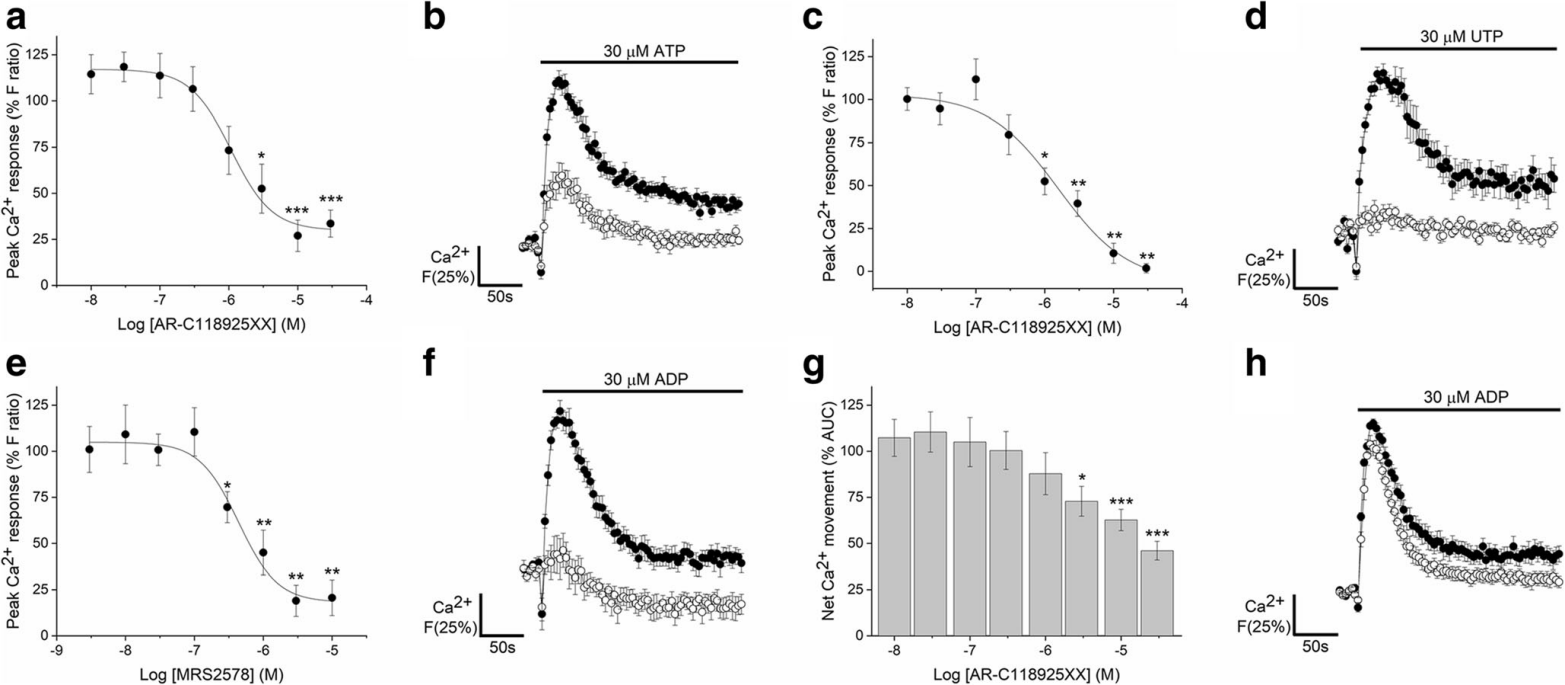
Effect of P2Y_2_ and P2Y_6_ receptor antagonism on ATP, ADP and UTP-elicited intracellular Ca^2+^ responses in human adipose-derived mesenchymal stromal cells. **a** Dose inhibition curve showing the effect of a selective P2Y_2_ receptor antagonist (AR-C118925XX) on intracellular Ca^2+^ responses elicited by 30 μM ATP (*N* = 7 donors). **b** Average time-resolved trace showing ATP-elicited Ca^2+^ response (30 μM) in the absence (*closed circles*) and presence (*open circles*) of AR-C118925XX (10 μM) (*N* = 7 donors). **c** Dose inhibition curve showing the effect of AR-C118925XX on intracellular Ca^2+^ responses elicited by 30 μM UTP (*N* = 7 donors). **d** Average time-resolved trace showing UTP-elicited Ca^2+^ response (30 μM) in the absence (*closed circles*) and presence (*open circles*) of AR-C118925XX (10 μM) (*N* = 7 donors). **e** Dose inhibition curve showing the effect of selective antagonism of P2Y_6_ receptors with MRS2578 on intracellular Ca^2+^ responses elicited by 30 μM ADP (*N* = 7 donors). **f** Average time-resolved trace showing ADP-elicited Ca^2+^ response (30 μM) in the absence (*closed circles*) and presence (*open circles*) of MRS2578 (10 μM). **g** Effect of AR-C118925XX on net Ca^2+^ movement evoked by ADP (30 μM) (*N* = 6 donors). **h** Average time-resolved trace showing ADP-elicited Ca^2+^ response (30 μM) in the absence (*closed circles*) and presence (*open circles*) of AR-C118925XX (10 μM). Data points are mean ± SEM. *p < 0.05, **p < 0.01, ***p < 0.001

Selective antagonists of P2X and P2Y receptors were employed to determine the molecular basis of the nucleotide-evoked responses. The ATP response was insensitive to selective antagonism of P2X4, P2X7, P2Y_1_, P2Y_11_, P2Y_12_ and P2Y_13_ receptors (*N =* 6) (Table 3). AR-C118925XX, a selective competitive P2Y_2_ receptor antagonist [22, 23], caused concentration-dependent inhibition of the peak response to ATP (IC_50_ 1.1 ± 0.8 μM, *N* = 7), reaching a plateau inhibition of 73.0 ± 8.5% on average in the presence of 10 μM antagonist (*N* = 7) (Fig. 5a–b). Net elevation in intracellular Ca^2+^ in response to ATP, as calculated by the area under the curve, was inhibited by 81.5 ± 3.3% at maximal concentrations of AR-C118925XX. Although AR-C118925XX had an inhibitory effect on the ATP response in all donors tested, there was some variation between donors. In three of the seven donors tested, the response to ATP was abolished in the presence of 3 μM antagonist. Furthermore, AR-C118925XX abolished UTP-evoked responses with the same potency as the ATP response (IC_50_ 1.6 ± 0.6 μM, *N* = 6) (Fig. 5c–d). Together with the observation that ATP and UTP are equipotent agonists in AD-MSCs, these data strongly suggest that the ATP and UTP responses are mediated by P2Y_2_ receptor activation.

**Table 3:** The effect of P2 subtype-selective antagonism on the nucleotide-evoked calcium responses in primary human adipose-derived mesenchymal stromal cells. The number of independent donor cells used (*N* number) is indicated within round brackets in the maximum inhibition and IC_50_ columns

Selective antagonist	Receptor target	Nucleotide [concentration, μM]	Antagonist range, μM	Maximum inhibition (%)^a^ [antagonist concentration, μM]	IC_50_, μM^a^	Reference
PSB-12062	P2X4	ATP [100]	0.003–30	ns	–	[57]
A438079	P2X7	ATP [1000]	0.003–10	ns	–	[58]
MRS2500	P2Y_1_	ATP [30]	0.0001–1	ns	–	[59]
		ADP [30]	0.0001–1	ns	–	
AR-C118925XX	P2Y_2_	ATP [30]	0.003–30	73.0 ± 8.5 [10] (7) 81.5 ± 3.3 [10] (5)	1.1 ± 0.8 (7) 2.9 ± 1.1 (5)	[22, 23]
		ADP [30]	0.003–30	18.2 ± 6.6 [30] (6) 53.9 ± 5.1 [30] (6)	0.64 ± 0.4 (6) 9.6 ± 1.6 (6)	
		UTP [30]	0.003–30	100 [30] (6) 100 [30] (6)	1.6 ± 0.6 (6) 0.84 ± 0.5 (6)	
MRS2578	P2Y_6_	ADP [30]	0.003–10	79.4 ± 9.6 [10] (6) 166.9 ± 27.3 [10] (6)	0.44 ± 0.1 (6) 0.29 ± 0.1 (6)	[24]
NF340	P2Y_11_	ATP [30]	0.003–10	ns	–	[60]
		ADP [30]	0.003–10	ns	–	
PSB-0739	P2Y_12_	ATP [30]	0.003–10	ns	–	[61]
		ADP [30]	0.003–10	ns	–	
MRS2211	P2Y_13_	ATP [30]	0.003–10	ns	–	[62]
		ADP [30]	0.003–3	ns	–	

^a^IC_50_ values and max % inhibition values were calculated with the peak magnitude values (top) and area under the curve data (bottom) for each agonist/antagonist combination. ns indicates no significant inhibition at any concentration of antagonist tested

ADP responses were insensitive to selective antagonism of P2Y_1_, P2Y_11_, P2Y_12_ and P2Y_13_ receptors (*N* = 6) (Table 3). However, MRS2587, a selective P2Y_6_ receptor antagonist [24], displayed potent antagonism (IC_50_ 437 ± 133nM, *N* = 6) of the ADP response and inhibited the peak response elicited by maximal ADP concentrations by > 80% (Fig. 5e–f). This antagonist also caused the concentration of intracellular Ca^2+^ to drop below baseline calcium post agonist stimulation. The only other antagonist to affect the response to ADP was AR-C118925XX, which did not have a significant effect on the magnitude of the response (≤ 10 μM AR-C118925XX), but caused the response to decay faster which attributed to a decrease in the net movement of Ca^2+^ by 37.3 ± 5.8% (*p* < 0.001, *N* = 6) (Fig. 5g–h).

### mRNA and protein expression of P2 receptors in MSCs

Analysis of mRNA transcripts revealed expression of P2X1, P2X4, P2X5, P2X6, P2X7, P2Y_1_, P2Y_2_, P2Y_4_ and P2Y_6_ receptors in MSCs (*N* = 6). However, there was heterogeneity in the expression of P2Y_11_, P2Y_12_, P2Y_13_ and P2Y_14_ receptors, as some donors had *C*_T_ values above 35 for these receptors (Table 4). In the case of the P2Y_12_ receptor, only two of the six donors tested had *C*_T_ values below 35, but even in these donors the average *C*_T_ value was 34.4 ± 0.3 (*N =* 2), indicating very low expression. P2X2 and P2X3 receptors were not expressed in any of the donors tested (*N* = 6) (Table 4).

**Table 4:** The mRNA expression profile of P2X and P2Y receptors in primary human adipose-derived mesenchymal stromal cells. Genes with *C*_T_ values of ≤ 35 were considered present and the average *C*_T_ values shown were calculated from *C*_T_ values below this threshold only. Cases of donor variation are indicated by ^a b c^. Data was collected for six independent donors and shown as mean ± SEM

Receptor	*C*_T_ value	Normalised to RPLP0 expression (∆C_T_)
P2X1	30.4 ± 0.4	11.3 ± 0.6
P2X2	Not detected	–
P2X3	37.8 ± 0.3	–
P2X4	25.8 ± 0.3	6.3 ± 0.6
P2X5	30.6 ± 1.3	12.8 ± 0.4
P2X6	32.9 ± 0.5	13.7 ± 0.7
P2X7	31.6 ± 0.2	12.1 ± 0.7
P2Y_1_	29.0 ± 0.8	11.2 ± 0.7
P2Y_2_	31.6 ± 0.5	13.8 ± 0.2
P2Y_4_	30.9 ± 0.5	13.1 ± 0.2
P2Y_6_	29.4 ± 0.8	10.4 ± 0.6
P2Y_11_	31.4 ± 0.7^a^	13.3 ± 0.8^a^
P2Y_12_	34.4 ± 0.3^b^	17.3 ± 0.6^b^
P2Y_13_	33.0 ± 0.6^c^	15.2 ± 0.5^c^
P2Y_14_	32.8 ± 0.5^c^	14.9 ± 0.4^c^

^a^Mean ± SEM for 4/6 donors

^b^Mean ± SEM for 2/6 donors

^c^Mean ± SEM for 5/6 donors

**Fig. 6:**
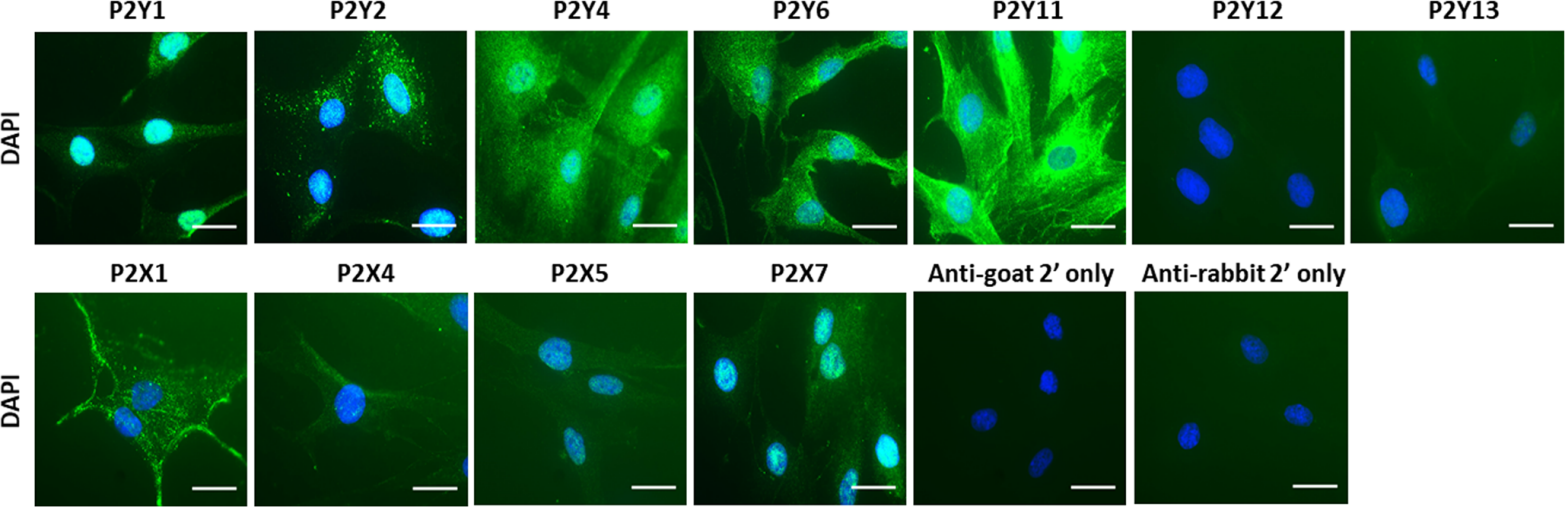
Analysis of P2Y and P2X receptor immunofluorescence in human adipose-derived mesenchymal stromal cells (MSCs). Images taken with a × 63 objective on a Zeiss AxioPlan 2ie epifluorescent microscope of permeabilised MSCs labelled with primary antibodies against receptor targets and visualised with an Alexa Fluor 488-conjugated secondary antibody (*green*). Cells are counterstained with DAPI to visualise nuclei (*blue*). The exposure and camera settings were consistent across all the images taken for each donor. Images presented are representative of at least ten fields of view of cells from three independent donors. Scale bar represents 30 μm

The protein expression of P2Y_1_, P2Y_2_, P2Y_4_, P2Y_6,_ P2Y_11_, P2Y_13_, P2X1, P2X4, P2X5 and P2X7 receptors was confirmed by immunocytochemistry. These P2 receptors appear to be distributed quite uniformly throughout the cytoplasm/cell membrane, with P2Y_1_ and P2X7 receptors also displaying staining in the nuclear/perinuclear regions. Very faint positive staining was observed for P2Y_13_ and P2X5 receptors. No staining was observed for the P2Y_12_ receptor (Fig. 6 and Online resource 2).

In summary, these results suggest that although a wide repertoire of P2 receptors were detected by qRT PCR and immunocytochemistry, only P2Y_2_ and P2Y_6_ receptors play a functional role in the nucleotide-evoked Ca^2+^ responses in MSCs.

## Discussion

This study provides clear evidence of functional P2 receptors in primary human AD-MSCs. Robust changes in intracellular calcium levels were observed with exogenous nucleotide stimulation of these cells. The abolishment of the ATP-elicited response by both emptying the ER calcium stores and inhibiting PLC, as well as the persistence of the response in the absence of extracellular calcium, suggests that the response is mediated by metabotropic P2Y receptors via the G_α__,q/11_-PLC-IP3R pathway. The potency of the response remains unchanged in the presence and absence of extracellular calcium, suggesting that the same receptors are likely to be activated in both cases. However, the magnitude of the response to ATP is diminished by the removal of extracellular calcium. This may be due to partial emptying of the intracellular calcium stores as the cell attempts to maintain calcium homeostasis without calcium influx, leading to less calcium being available to respond to receptor activation. Alternatively, the magnitude of the response may be dependent on extracellular Ca^2+^ entry as well as Ca^2+^ release from intracellular stores. Removal of extracellular Ca^2+^ eliminates the lingering increase in intracellular Ca^2+^ evoked by nucleotide application, which may indicate that this latter plateau phase is due to store-operated Ca^2+^ entry (SOCE). Purinergic signalling via P2Y receptors has previously been shown to lead to SOCE [25, 26].

The presence of a UTP-evoked calcium response in human AD-MSCs may be indicative of P2Y_4_ receptor involvement. Unfortunately, there is currently no commercially available selective antagonist for P2Y_4_ receptors [27], so it is not possible to conclusively eliminate the possibility that P2Y_4_ receptors may have a role in the UTP-evoked calcium response; however, the evidence presented here strongly supports the hypothesis that the ATP and UTP responses are mediated by P2Y_2_ receptors. The calculated EC_50_ values for ATP and UTP and the IC_50_ values in the presence of a selective P2Y_2_ receptor antagonist are comparable, which fits with reports that ATP and UTP act equipotently on P2Y_2_ receptors [28], whereas P2Y_4_ receptors are antagonised by ATP [29, 30]. In addition, the ATP responses are blocked and the UTP responses are abolished by a P2Y_2_ receptor-selective antagonist, which strongly suggests P2Y_2_ receptor involvement, and precludes P2Y_4_ receptor contribution. It has recently been suggested that the P2Y_2_ receptor may play a role in driving BM-MSC adipogenesis while suppressing osteogenesis, without effecting the rate of cell proliferation [31]. It may be that P2Y_2_ receptors are involved in a similar role in AD-MSCs. If this was the case, this could provide an opportunity to develop pharmacological tools to target the P2Y_2_ receptor specifically to drive MSCs towards/away from an adipogenic phenotype in vivo, thus providing a route to control the number of new adipocytes present in adipose tissue and potentially regulate weight gain.

It is important to note that although selective P2Y_2_ receptor antagonism abolishes the response to UTP, approximately 25% of the peak ATP response is resistant to the effects of AR-C118925XX. This residual response may be simply due to the competitive nature of the antagonist or due to the liberation of ADP (and/or AMP and adenosine) from ATP by ectonucleotidases, such as CD39 [32, 33], resulting in the activation of other P2 receptors. This may also explain the variation in the level of inhibition observed between donors, which may be due to the differing efficiencies of ectonucleotidase activity per donor. No genetic analysis of the donors used in this study was conducted, but previous reports suggest that mutations in the *ENTPD1* (CD39) gene can lead to a reduction in the activity of CD39 [34] and there have also been reports to suggest that CD39 surface expression levels are dynamic and can increase under certain conditions [35].

A robust ADP response was resistant to inhibition in the presence of all the antagonists tested, excluding AR-C118925XX (P2Y_2_) and MRS2578 (P2Y_6_). AR-C118925XX only significantly inhibited the net movement of Ca^2+^ in the latter decay phase of the response to ADP and not the initial peak. ADP is not the preferred agonist for P2Y_2_ receptors, so these results may indicate indirect activation of the P2Y_2_ receptor via ADP-induced release of ATP [26]. The ADP response was also inhibited by MRS2578. ADP has been shown to elicit a calcium response in 1321N1 astrocytoma cells over-expressing P2Y_6_ receptors, but its effects are much less potent than the preferred agonist of P2Y_6_ receptors, UDP [36], so it was surprising that MRS2578 abolished the response to ADP in MSCs, when these cells lack a UDP-elicited Ca^2+^ response. It is important to note that UDP does elicit a Ca^2+^ response in MSCs, but only at very high agonist concentrations that are more than ten-fold higher than concentrations required to maximally active the P2Y_6_ receptor [36]. One possible explanation for this may be that P2Y_6_ receptors exist as a heterodimer, which has led to an altered agonist profile in these cells. P2Y_6_ receptors have been shown to form heterodimers with other GPCRs [37, 38] and hetero-oligomerization of other P2Y receptors can alter the agonist sensitivity of these receptors [39]. After the initial peak, blockade of the ADP response with MRS2578 leads to a decrease in the concentration of intracellular Ca^2+^ to below baseline Ca^2+^ levels, suggesting there is either efflux or internalisation of Ca^2+^. It is unclear why P2Y_6_ receptor inhibition would have this effect and these observations imply that the role of the P2Y_6_ receptor in MSCs is complex. P2Y_6_ receptors have been shown to promote osteogenesis in BM-MSCs [40] and has also been implicated in increasing IL-6 expression [41]. It has been suggested that IL-6 is important for maintaining the immunoprivilege stasis of MSCs [42], so if P2Y_6_ receptors are involved in IL-6 secretion it may prove to be a valuable target for prolonging MSC viability for therapeutic use. However, much more additional work is required to clarify both the molecular mechanism and functional role of P2Y_6_ receptor activation in these cells.

Despite evidence to suggest the presence of P2X receptors in AD-MSCs, no functional evidence for ionotropic involvement in the nucleotide-evoked Ca^2+^ responses was observed. A previous study conducted by Zippel et al. (2012) also detected P2X receptors in AD-MSCs, but interestingly although they did not detect P2X1 receptor expression at the mRNA level, they demonstrated that use of P2X1 receptor-selective antagonist, NF279, was able to inhibit the ATP-evoked calcium response [43]. This discrepancy may be explained by the fact they used extremely high concentrations of NF279 (100 μM) which is likely to non-specifically block other P2 receptors as well [44]. P2X4 receptors are known to localise to lysosomes and traffic to and from the plasma membrane in other cell types [45, 46], so one explanation for the lack of effect of P2X4 receptor antagonism is that very few P2X4 receptors are expressed at the cell surface. Other groups have demonstrated presence of P2X7 receptors in mesenchymal stem cells [47], but in this study, we were unable to demonstrate functional P2X7 receptors, possibly due the use of insufficiently elevated concentrations of ATP in this study or the unusual localisation of the P2X7 receptor in the nuclear/perinuclear region. P2Y_1_ and P2Y_11_ receptors were also detected at the mRNA and protein level, but selective antagonism of these receptors did not suggest a role for either receptor in the nucleotide-evoked Ca^2+^ responses in these cells. A possible explanation for this is that P2Y_2_ receptor activation caused heterologous desensitisation of these subtypes [48]. As well as being found throughout the cell, like the P2X7 receptor, the P2Y_1_ receptor also appears to be located in the nuclear/perinuclear region, and this may be a reason for the lack of P2Y_1_ receptor involvement in the response to exogenous nucleotide application. It is unclear why these receptors are detected close to the nucleus in these cells. Antagonism of the G_i_-coupled P2Y_12_ and P2Y_13_ receptors had no effect on the Ca^2+^ response. P2Y_12_ receptors do not appear to be expressed at the protein level in these cells.

It must be acknowledged that there are several P2 receptors that were detected at the mRNA level, but were excluded from further study. Current evidence suggests both P2X5 and P2X6 receptors are non-functional in humans [49–51]. The response to the P2X1 receptor rapidly desensitises, so it is unlikely to be detected with a FlexStation III. Finally, there was no functional Ca^2+^ response detected at physiologically relevant concentrations of UDP-glucose, the preferred agonist of P2Y_14_ receptors. Responses to UDP-glucose were only detected at very high concentrations of agonist, but previous reports suggest that 10 μM UDP-glucose is sufficient to maximally activate P2Y_14_ receptors [52].

It must also be noted that there are several limitations associated with this study. Firstly, only female patients were recruited and no information about ethnicity or lifestyle was collected. The mean age of the recruited donors is 55.5 ± 1.4, and ageing has been shown to increase cellular senescence and AD-MSCs extracted from elderly patients (age 60–73) have been shown to have diminished migration and differentiation capabilities [53]. The molecular mechanisms underlying these changes are not fully understood, so it is not possible to rule out potential changes in the P2 receptor expression and/or functioning as a possible cause. However, data presented by Zippel et al. (2012) included younger donors and they demonstrated functional response to both ATP and UTP in AD-MSCs extracted from younger patients and they also detected a very similar P2 receptor profile to the profile determined in this study [43]. However, it is important to note that Zippel et al. (2012) only used cells isolated from a total of three donors, so a much more extensive study would be required to clarify whether the results presented here are applicable to younger donors as well. This means the data presented here is limited to profiling the P2 receptor responses in AD-MSCs extracted from older women. Also, although all the recruited patients were cancer-free at the time of recruitment, each donor has been treated for breast cancer in the past. Although previous reports suggest some chemotherapeutic agents do not affect surface marker expression, proliferation and differentiation of AD-MSCs [54], it is not known whether cancer and/or subsequent cancer treatment causes long-term alterations in the expression and function of purinergic receptors in these cells. Perhaps the most important limitation of the study is the use of an in vitro system. Cell culture has been previously shown to cause changes in the behaviour of MSCs [8, 55, 56], so this in vitro model may not accurately reflect conditions in vivo. This study depends heavily on the selectivity of available pharmacological probes and antibodies. Although all the pharmacological antagonists used in this study are reported to be selective for their target receptor, it is not possible to rule out the possibility that these antagonists affect non-specific targets in these cells. Finally, all the pharmacological data presented here is based on analysis of population level response, which does not account for cell-to-cell variation in response patterns. Single-cell calcium imaging would be a good method to investigate any heterogeneity within the population.

In summary, we have demonstrated that all the known P2 receptors are expressed in human AD-MSCs, excluding P2X2, P2X3 and P2Y_12_ receptors, but some heterogeneity in the expression of P2Y_11_, P2Y_13_ and P2Y_14_ receptors exists. Despite confirmation of the presence of receptors at both the mRNA and protein level, only two P2 receptors, P2Y_2_ and P2Y_6_, appear to be involved in generating functional nucleotide-evoked Ca^2+^ responses. It is likely that the other P2 receptors play a functional role in these cells, but this study suggests that they are not primarily involved in changing intracellular Ca^2+^ levels in response to exogenous nucleotide stimulation. They may be involved in non-Ca^2+^-dependent pathways, only activated under certain circumstances or have intracellular functions which are beyond the scope of this study. There has already been a lot of research into the role of P2 receptors in BM-MSCs, which suggests that P2Y_2_ and P2Y_6_ receptors are important in driving adipogenic and osteogenic differentiation respectively [20, 31, 40]; it is possible that these receptors may play a similar role in AD-MSCs as well. If so, targeting these receptors to drive adipogenesis may provide a novel method of controlling adipose tissue expansion and weight gain and/or help in efforts to improve the therapeutic potential of MSCs. Much more research is needed to elucidate the role of these receptors in vivo, but this study provides a solid foundation from which to begin.

## Electronic supplementary materials

ESM 1 (DOCX 3274 kb)

ESM 2 (DOCX 1764 kb)
