# Supplementary material for: P2Y2 and P2Y6 receptor activation elicits intracellular calcium responses in human adipose-derived mesenchymal stromal cells
Source: Purinergic Signal. 2018 Aug 7;14(4):371–84. doi: 10.1007/s11302-018-9618-3 (PMC6298923; doi:10.1007/s11302-018-9618-3)
Supplement: Supplementary file 1 — (DOCX 3274 kb) [file 11302_2018_9618_MOESM1_ESM.docx]

**Supplementary Information – Purinergic Signalling**

**P2Y_2_ and P2Y_6_ receptor activation elicits intracellular calcium responses in human adipose-derived mesenchymal stromal cells.**

Seema Ali^1^, Jeremy Turner^2,3^ and Samuel J. Fountain^1^

^1^ School of Biological Sciences, University of East Anglia, Norwich, NR4 7TJ, UK.

^2^ Norfolk & Norwich University Hospital, Norwich, NR4 7UY, UK.

^3^ Norwich Medical School, University of East Anglia, Norwich, NR4 7TJ, UK.

**CORRESPONDING AUTHOR’S DETAILS**

Dr Samuel J. Fountain

School of Biological Sciences, University of East Anglia, Norwich Research Park, Norwich, NR4 7TJ, UK

Email address: [s.j.fountain@uea.ac.uk](mailto:s.j.fountain@uea.ac.uk)

Telephone: +44 (0)1603 597326


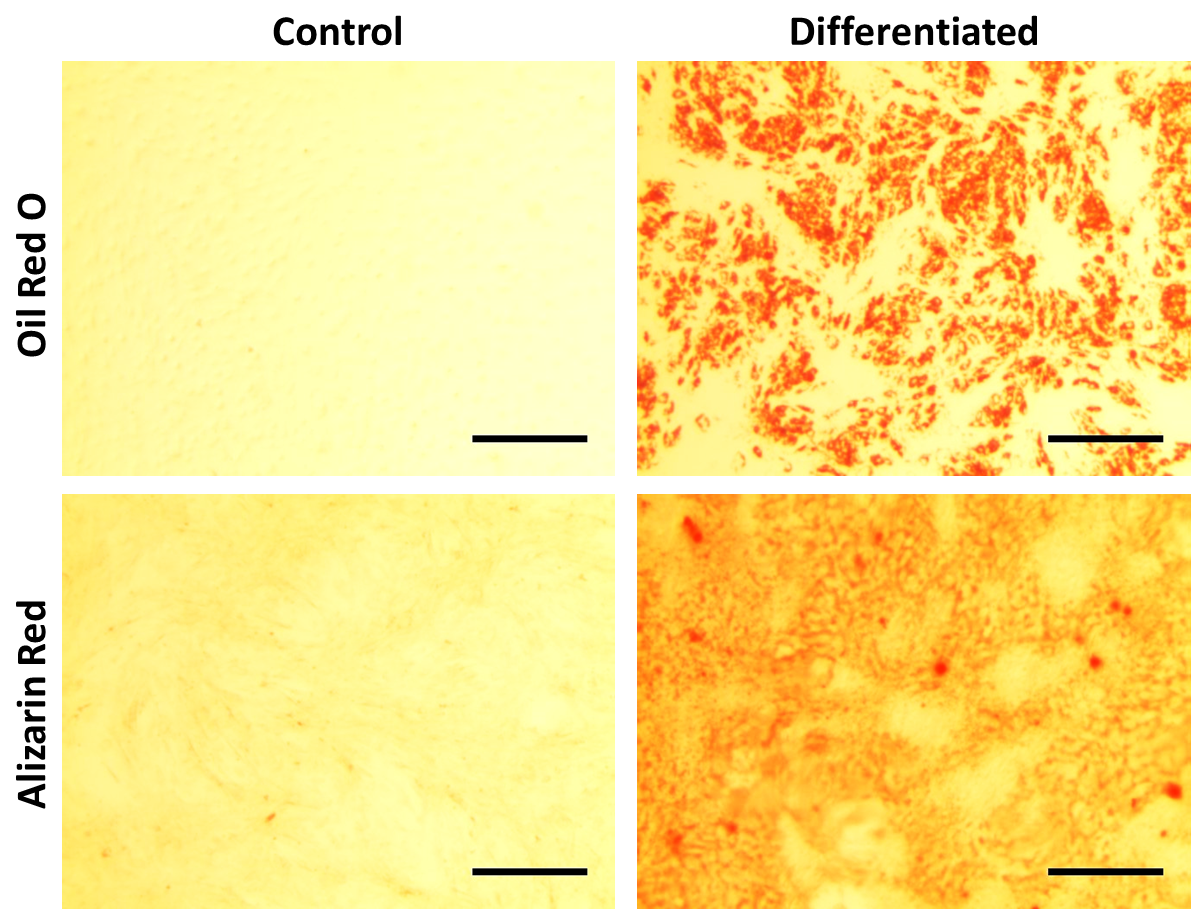


**Supplementary Figure 1**. Primary human adipose-derived mesenchymal stromal cells can be induced to differentiate to adipocytes and osteoblasts, but no spontaneous differentiation occurs. Cells were cultured in the presence of culture media (controls, left column), adipogenic media (top right) or osteogenic media (bottom right) for two weeks and then the adipocytes were stained with Oil Red O and the osteoblasts were stained with Alizarin Red. Scale bar represents 150 μm.

**Methods:**

**Differentiation of mesenchymal stromal cells to adipocytes or osteoblasts**

Human adipose-derived mesenchymal stromal cells (MSCs) were seeded in to plastic or glass-bottom plates/flasks and grown to hyper-confluency, by incubating the cells are 37 ^o^C + 5% CO_2_ for four days. The culture media was then removed and replaced with adipogenic or osteogenic media. Adipogenic media consisted of culture media (DMEM, 10% FBS (v/v) and 50 IU/ml penicillin, 50 µg/ml streptomycin) supplemented with 100 nM insulin, 1 μM dexamethasone, 200 μM indomethacin and 500 μM 3-isobutyl-1-methylxanthine, whereas osteogenic media consisted of culture media supplemented with 10 mM sodium β-glycerophosphate, 50 μg/ml L-ascorbic acid-2-phosphate and 100 nM dexamethasone. In both cases, the cells were left in the presence of the differentiation media for two weeks and during that period the media was replaced with fresh media every four days. After two weeks, the media was removed and replaced with ‘normal’ culture media for three days to allow the cells to habituate. Then the cells were ready for experimentation.

**Oil red O staining**

Human adipose-derived mesenchymal stromal cells (MSCs) were washed with 1X PBS once and then fixed with 4% paraformaldehyde (PFA) for 5 minutes at room temperature. The cells were then washed once with distilled water and incubated with 60% isopropanol. The isopropanol was then removed and replaced with oil red O dye and the cells were incubated with oil red O for 5 minutes at room temperature. The dye was then removed and the cells were washed with PBS and visualised using a light microscope. Images were taken using an Olympus CKX41 inverted microscope (Olympus, Tokyo, Japan) using a 2.5X objective.

**Alizarin red staining**

Human adipose-derived MSCs were washed with PBS lacking Ca^2+^ and Mg^2+^ and then fixed with 4% paraformaldehyde (PFA) for 15 minutes at room temperature. Then the cells were washed with deionised water once and then incubated with alizarin red S dye (pH 4.1) for 45 minutes at room temperature in the dark. The dye was then removed and the cells were washed four times with deionised water and the cells were visualised using a light microscope. Images were then taken using an Olympus CKX41 inverted microscope (Olympus, Tokyo, Japan) using a 2.5X objective.
