# Supplementary material for: P2Y2 and P2Y6 receptor activation elicits intracellular calcium responses in human adipose-derived mesenchymal stromal cells
Source: Purinergic Signal. 2018 Aug 7;14(4):371–84. doi: 10.1007/s11302-018-9618-3 (PMC6298923; doi:10.1007/s11302-018-9618-3)

**Supplementary Information 2 – Purinergic Signalling**

**P2Y_2_ and P2Y_6_ receptor activation elicits intracellular calcium responses in human adipose-derived mesenchymal stromal cells.**

Seema Ali^1^, Jeremy Turner^2,3^ and Samuel J. Fountain^1^

^1^ School of Biological Sciences, University of East Anglia, Norwich, NR4 7TJ, UK.

^2^ Norfolk & Norwich University Hospital, Norwich, NR4 7UY, UK.

^3^ Norwich Medical School, University of East Anglia, Norwich, NR4 7TJ, UK.

**CORRESPONDING AUTHOR’S DETAILS**

Dr Samuel J. Fountain

School of Biological Sciences, University of East Anglia, Norwich Research Park, Norwich, NR4 7TJ, UK

Email address: [s.j.fountain@uea.ac.uk](mailto:s.j.fountain@uea.ac.uk)

Telephone: +44 (0)1603 597326

**Supplementary Figure 2. P2 purinergic receptors immunofluorescence in human adipose-derived mesenchymal stromal cells (MSCs).** Images taken with a 10x objective on a Zeiss AxioPlan 2ie epifluorescent microscope of permeabilised MSCs labelled with primary antibodies against receptor targets and visualized with an Alexa Fluor 488-conjugated secondary antibody (*green*). Cells were counterstained with DAPI to visualize nuclei (*blue*). The exposure and camera settings were consistent across all the images taken for each donor. Images presented are representative of at least ten fields of view for three independent donors. Scale bar represents 200 μm.


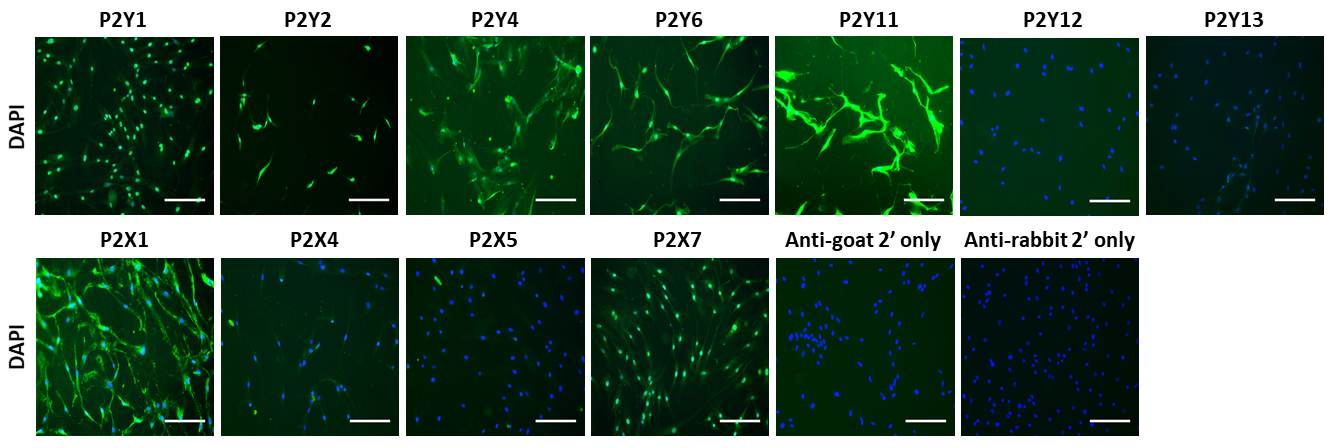

Supplement: Supplementary file 2 — (DOCX 1764 kb) [file 11302_2018_9618_MOESM2_ESM.docx]
